# Quality of Life in Women with Deep Endometriosis: A Cross-Sectional Study

**DOI:** 10.1055/s-0040-1708091

**Published:** 2020-02

**Authors:** Daniela Angerame Yela, Iuri de Paula Quagliato, Cristina Laguna Benetti-Pinto

**Affiliations:** 1 Department of Gynecology and Obstetrics, School of Medical Sciences, Universidade de Campinas, Campinas, SP, Brazil

**Keywords:** endometriosis, quality of life, pelvic pain, dysmenorrhea, endometriose, qualidade de vida, dor pélvica, dismenorreia

## Abstract

**Objective** To describe clinical and sociodemographic characteristics of women with deep infiltrating endometriosis (DIE) and assess their quality of life (QOL) during 6 months of medical treatment.

**Methods** A descriptive cross-sectional study of 60 women diagnosed with DIE either by surgery or image methods (ultrasound or magnetic resonance), who received clinical treatment for at least 6 months in the Universidade de Campinas, Campinas, state of São Paulo, Brazil. Both the SF-36 and the EHP-30 questionnaires were used to assess the quality of life.

**Results** The mean age of the patients was 37.7 ± 6.0 years old, with 50% presenting dysmenorrhea; 57% dyspareunia; and 50% chronic pelvic pain. The SF-36 and the EHP-30 revealed impaired quality of life. In the SF-36, the worst domains were limitation due to emotional aspects (40.2 ± 43.1) and self-esteem and disposition (46.1 ± 24.8), whereas in the EHP-30 they were social well-being (50.3 ± 30.6); infertility (48.0 ± 36.3); and sexual intercourse (54.0 ± 32.1).

**Conclusion** Although clinically treated, women with deep endometriosis present impairment in different domains of quality of life regardless of the questionnaire used for evaluation.

## Resumo

**Objetivo** Descrever características clínicas e sociodemográficas de mulheres com endometriose profunda infiltrativa e avaliar sua qualidade de vida dentro de 6 meses de tratamento clínico.

**Métodos** Estudo de corte transversal descritivo com 60 mulheres em seguimento ambulatorial na Universidade de Campinas, Campinas, SP, Brasil, com endometriose profunda infiltrativa diagnosticada por cirurgia ou métodos de imagem (ultrassonografia ou ressonância magnética), em tratamento clínico há pelo menos 6 meses. Para avaliar a qualidade de vida, foram utilizados os questionários SF-36 e EHP-30.

**Resultados** A média etária das mulheres foi de 37,7 ± 6,0 anos; 50% delas apresentaram dismenorreia, 57% dispareunia e 50% dor pélvica crônica. O SF-36 e o EHP-30 mostraram comprometimento da qualidade de vida destas mulheres. No SF-36, os piores domínios foram os aspectos emocionais (40,2 ± 43,1) e a autoestima e disposição (46,1 ± 24,8), enquanto que no EHP-30 foram o bem-estar social (50,3 ± 30,6), a infertilidade (48,0 ± 36,3) e as relações sexuais (54,0 ± 32,1).

**Conclusão** Embora tratadas clinicamente, as mulheres com endometriose profunda apresentaram comprometimento em diferentes domínios da qualidade de vida independente do questionário utilizado para avaliação.

## Introduction

Endometriosis is characterized by the presence of endometriotic tissue beyond the uterine cavity, mainly in the ovaries and other pelvic organs.^1^ Women with endometriosis may be asymptomatic or may report symptoms of dysmenorrhea; deep dyspareunia; chronic pelvic pain; urinary pain or intestinal pain; and infertility.^2^

Although all types may cause pain, deep lesions are usually related to a more severe clinical status, impairing quality of life (QOL); daily work activities; social relationships; and frequently the fertility of these women.^3^ ^4^ ^5^ Studies report a reduction in ∼ 38% of the work productivity of these women, mainly attributed to pelvic pain.^4^ Furthermore, ∼ 88% of these women had anxiety disorders or depression.^6^ The disease was also highly relevant in the area of human reproduction, since 50% of patients diagnosed with endometriosis had some fertility disorder, due to chronic inflammation and the formation of pelvic adhesions.^7^

Studies have shown that endometriosis has a negative impact on QOL.^4^ ^8^ ^9^ However, with surgical and/or medical treatment, QOL is improved.^10^ ^11^ ^12^ ^13^ ^14^ The majority of studies in the literature did not evaluate women according to the disease stage, although few studies show a poorer QOL in later stages.^15^ In addition, women with chronic pain have a lower QOL due to both physical and psychological factors.^16^ ^17^ The literature also shows that pain reduction is generally not related to the improvement of psychological disturbances.^18^ A recent study with women with deep endometriosis has shown that it affects these women globally and that more studies are needed to better understand the whole context.^19^ Therefore, because endometriosis can negatively affect QOL, particularly in more advanced stages, the main goal of the present study is to assess QOL in women with deep infiltrating endometriosis (DIE).

## Methods

A descriptive cross-sectional study was performed with 60 women undergoing medical treatment for at least 6 months at the Universidade de Campinas (UNICAMP, in the Portuguese acronym), Campinas, State of São Paulo, Brazil, for DIE, diagnosed surgically or by imaging (nuclear magnetic resonance imaging [NMRI] or transvaginal ultrasound with bowel preparation). Deep infiltrating endometriosis was characterized when imaging exams showed lesions in the retrocervical region (uterosacral ligaments and uterine torus), the vagina, the intestine (rectum, sigmoid, ileum and appendix), the bladder and the ureter. Women who were surgically diagnosed had performed the procedure for > 2 years and had recurred from the injuries after surgery. Those included in the study were exclusively on drug treatment. Women with cognitive impairment, incapable of understanding the instruments, and women with other chronic diseases such as neoplasias; lower back pain; psychiatric disorders; rheumatologic diseases; among others that could impact the QOL, were excluded.

The diagnosis of DIE was considered surgical when a surgical description of deep endometriosis was presented along with a confirmatory anatomopathological report (20 women had surgical diagnosis). All of the women with surgical diagnosis were on hormonal treatment to prevent recurrence and to control pain. Clinical diagnosis was performed by transvaginal ultrasound with bowel preparation or NMRI (40 women had imaging diagnosis). The diagnosis of deep endometriosis established by imaging was performed through transvaginal sonography with bowel preparation or NMRI, always performed by the same expert during the diagnosis. All of the women had pain symptoms and 20 women had infertility, and at the time of the interview 9 were asymptomatic.

The variables analyzed were: QOL; age; color (white, non-white); school education (illiterate, elementary school level, high school level, university level); family income; professional activity (unemployed, employed); marital status (with a partner, without a partner); religion (atheist, catholic, protestant and other); number of pregnancies; parity; body mass index (BMI) (calculated by weight in kilograms divided by height in square meters, classified as underweight < 20kg/m^2^, normal 20 to 25kg/m^2^, overweight 25 to 30kg/m^2,^ and obese ≥ 30kg/m^2^); treatment used, symptoms of endometriosis (dysmenorrhea, dyspareunia, chronic pelvic pain, pain to urinate and pain to defecate), smoker (yes or no), surgical history (any previous surgeries such as abdominal surgeries or cesareans). Treatments used for pain control were hormonal (progestin alone – 53 women, or combined oral contraceptives – 5 women) or non-hormonal (nonsteroidal anti-inflammatory agents – 2 women).

Pain symptoms were evaluated according to the pain visual analog scale (VAS) on a scale of 0 to 10, where 0 represented absence of pain and 10 indicated maximum pain. Pain was classified as mild when the score was 1 to 3, moderate when the score was 4 to 6 and severe when it was > 7.^20^

To assess QOL, two questionnaires were used: the Endometriosis Health Profile Questionnaire (EHP-30) and the Short Form – 36 (SF-36). The EHP-30 questionnaire was developed by Jones et al, in 2001^21^ and was validated to Brazilian Portuguese in 2008 by Mengarda et al.^22^ It consists of 30 items covering five dimensions: pain; control and capacity to cope with the disease (hopelessness); emotional well-being; social support and self-image; and a modular questionnaire with 23 items distributed in six scales: sexual intercourse; work; doctor x patient relationship (medical profession); infertility; relationship with children and treatment. Each item is assessed on a 4-point scale (never = 0, rarely = 1, sometimes = 2, many times = 3, always = 4). Scoring is transformed into a 0 to 100 scale, where the lowest score means a better QOL.

The SF-36 questionnaire was developed by Ware et al in 1992^23^ and was validated to Brazilian Portuguese in 1999 by Ciconelli et al.^24^ It assesses 8 dimensions: functional aspects; physical aspects; pain; general health status; vitality; social aspects; emotional aspects; and mental health. It presents a final score of 0 to 100, in which zero corresponds to a worse general health status and 100 to a better general health status. Among the eight scales, three (functional capacity, physical aspects and pain) correspond to the Physical Component Summary (PCS), and the Mental Component Summary (MCS) includes mental health scales, emotional and social aspects. Two of the scales (vitality and general health) do not correlate with either component.^23^

All of the participants in the study signed an informed consent agreement. The present study was approved by the Research Ethics Committee of the Institution (number: 43715915.4.0000.5404).

### Statistical Analysis

To calculate the sample size, the method used to compare the average QOL score between 2 evaluations in a longitudinal study (before and after intervention) with a quantitative variable, was according to Browner et al,^25^ setting the significance level of α or type I error at 5% (α = 0.05) (or 95% confidence interval [CI]), and sample power at 80% (or 20% type II error [β = 0.20]), and obtaining the mean and standard deviation (SD) values of delta (difference of scores between before and after intervention) from a literature study.^26^ It was estimated that a sample of 63 women would be representative to compare the physical QOL score after 6 months of treatment, with a 5% significance level and 80% sample power.

Descriptive analysis expressed in relative frequencies was used for statistical analysis, for the categorical variables and for the continuous variables the analysis was expressed in mean and SD.

## Results

We evaluated 60 women treated clinically, out of which 2 were excluded due to the use of anti-inflammatory treatment, leaving 58 women who underwent hormonal clinical treatment.

The mean age of the patients was 37.2 ± 6.0 years old (minimum: 26 years old and maximum: 49 years old); 55% were nulliparous and 75% of the cases had already undergone previous surgery. Regarding clinical factors, 91% were undergoing progestin treatment; 51% had chronic pelvic pain; 58% had dyspareunia; 53% had dysmenorrhea, 13% had pain on urination and 43% had pain when defecating (Table 1). Regarding pain intensity, we observed that women reported scores of 7.5 ± 4.9 for dysmenorrhea, 7.4 ± 4.9 for dyspareunia, 7.2 ± 4.4 for chronic pelvic pain, 6.6 ± 4.9 for pain when defecating and 5.7 ± 0.7 for pain when urinating.

**Table 1:** Percentage distribution of women with deep infiltrating endometriosis according to sociodemographic and clinical characteristics (*n* = 58)

Characteristic	*n* (%)
Color	
White	44 (75.86)
Non-white	14 (24.14)
Marital status	
With a partner	37 (63.79)
Without a partner	21 (34.21)
Profession	
Employed	15 (25.86)
Unemployed	43 (74.14)
Education	
Illiterate	1 (1.73)
Elementary school level	14 (24.14)
High school level	28 (48.27)
University level	15 (25.86)
Gestation	
Nulliparous	32 (55.17)
Multiparous	26 (44.83)
BMI (kg/m^2^)	
≤ 20	4 (6.89)
20 -25	24 (41.39)
25–30	16 (27.58)
≥ 30	14 (24.14)
Smoking	
Yes	5 (8.63)
No	53 (91.37)
Previous surgery	
Yes	44 (75.86)
No	14 (24.14)
Treatment	
Progestin	53 (91.37)
Estrogen with progestin	5 (8.63)
Pain symptoms	
Dysmenorrhea	31 (53.44)
Chronic pelvic pain	30 (51.72)
Dyspareunia	34 (58.62)
Intestinal pain	25 (43.10)
Urinary pain	8 (13.79)

Abbreviation: BMI, body mass index.

The SF-36 exhibited median scores for all domains. The women had scores ≥ 50 on average in the physical domains of the SF-36, such as functional capacity; limitation due to physical aspects; pain; and general health status. Psychological domains (self-esteem 46.1 ± 24.8; disposition and emotional aspects 40.2 ± 43.1) affected the QOL in these women more intensely; although other psychological domains, such as social aspects and mental health, scored > 50 (Table 2).

**Table 2:** Descriptive analysis of questionnaire domains SF-36

Domains	*n*	mean	SD
Functional capacity	58	56.2	23.7
Limitation due to physical aspects	58	50.0	42.9
Pain	58	55.1	59.4
General health status	58	60.8	22.4
Self-esteem and disposition	58	46.1	24.8
Social aspects	58	57.8	29.5
Limitation due to emotional aspects	58	40.2	43.1
Mental health	58	51.1	24.0

Abbreviation: SD, standard deviation.

In the EHP-30, some questions from the domains work; children; sexual intercourse; doctor patient relationship; treatment and infertility were not answered by all of the women, because they did not feel empowered to respond (they had not experienced the questions asked or felt constrained in responding).

The EHP-30 showed a greater impairment in QOL in social domains (social well-being 50.3 ± 30.6; social support 48.0 ± 36.2), capacity to cope with the disease (44.7 ± 33.7) and fertility (48.0 ± 36.3). These women had a good doctor-patient relationship (18.1 ± 23.8). Only 21 out of 58 women responded to the children domain, since these patients were either nulliparous or felt uncomfortable talking about the subject (Table 3).

**Table 3:** Descriptive analysis of questionnaire domains EHP-30

Domains	*n*	Mean	SD
Pain	58	40.4	31.1
Control and capacity to cope with the disease	58	44.7	33.7
Social well-being	58	50.3	30.6
Social support	58	48.0	36.2
Self-image	58	41.2	34.2
Work	47	27.5	31.4
Children	21	33.9	37.3
Sexual intercourse	52	54.0	32.1
Doctor x patient relationship	56	18.1	23.8
Treatment	51	35.3	31.1
Infertility	39	48.0	36.3

Abbreviation: SD, standard deviation.

## Discussion

Assessment of QOL, by applying questionnaires, allows the measuring of the general impact of chronic diseases, such as endometriosis, on the lives of women. Two questionnaires were used in the current study, generic and specific, the SF-36 and the EHP-30, respectively, to observe whether the results were similar. The decision to apply both questionnaires is based on the fact that both address physical (pain) and psychological aspects that can influence the QOL in women with a chronic disease. However, the EHP-30 addresses some specific aspects of endometriosis that affect the QOL of these women, such as their sexual function; the question of motherhood; the relationship between doctor and patient; and the treatment of the disease. A recent review shows that endometriosis affects negatively the QOL of women with endometriosis but there is still no consensus on what would be the best instrument to evaluate it.^27^

In a review published in 2017 that evaluated 26 studies on endometriosis and QOL, only one used the EHP-30 and SF-36 questionnaires.^28^ And recently, a Brazilian study addressed QOL in women with deep endometriosis using the two questionnaires.^18^

Approximately half of the women in the study had good pain control, but those with this symptom had moderate to severe pain. Furthermore, on evaluation of their QOL, these women had better results in the physical domains than in the psychological domain. It is also important to highlight that these women had deep endometriosis. In the literature, studies usually do not assess QOL according to disease stage.

Studies in the literature have shown that women with a more severe pain level have a worse QOL, although physical QOL is only related to physical pain. According to models of mediation, increasing physical pain and difficulties in emotion regulation result in increased psychological stress, which is associated with deterioration in QOL. The main goal of the treatment is to reduce pain symptoms and improve QOL, decreasing the societal burden and health care costs related to endometriosis.^27^ ^29^

A review of 18 articles published in the past 5 years showed that understanding by the part of family members and partners may help women cope with this disease. In addition, pharmacological and surgical treatment may contribute toward pain control. However, other alternatives have been associated with the therapeutic management of endometriosis, such as lifestyle changes; physical exercise; diet; and sleep.^30^

A Brazilian study was performed in 2008 to investigate the relationship between clinical aspects and QOL in a group of 130 women with endometriosis. Data was collected using the SF-36 questionnaire, showing that patients with endometriosis had lower QOL scores than the general population.^27^

The term QOL should encompass three main dimensions: mental health; physical; and social functioning. Both questionnaires addressed these issues and apparently presented similar results. It has been observed that medical treatment helps a woman to improve in some items, since it was effective in pain control. Although management is not effective at obtaining an excellent QOL, it still exerts a significantly negative impact on QOL.

Studies have emphasized the central role of pain associated with endometriosis, which is negatively correlated with QOL; sexual functioning; quality of relationship with partner; mood; work; and social role functioning. Chronic pain may result in social isolation and can also negatively affect emotional well-being. Endometriosis is associated with psychological problems; anxiety; depression, and a weak capacity to cope with difficulty. It is noteworthy that there is a positive relationship between the level of anxiety symptoms and pain intensity. A decrease in the level of depressive symptoms is observed after pain treatment.^28^ Furthermore; it is worth mentioning that women with asymptomatic endometriosis may not report impairment of QOL and mental health.^31^ Another study showed greater improvement in physical domain scores than in those of mental domains for patients with deep endometriosis.^11^

In the SF-36 questionnaire, the domain due to emotional aspects and self-esteem/disposition had a mean score of 40.2 and 46.1, respectively, once again pointing to the mental health issue, which is not frequently addressed during medical consultations. Another Brazilian study of 128 women performed in 2007 suggested that psychological support with group intervention was a good adjunct to conventional treatment, since scores of pain and emotional aspects decreased.^32^ A recent study shows that women with deep endometriosis have worse physical and mental QOL than women with rheumatoid arthritis.^33^

The limitation of the present study is not having included a control group in the analysis. The literature shows that medical or surgical treatment may improve pain over time. The QOL in a woman with deep endometriosis is still unsatisfactory and inferior in comparison to the QOL of the general population, since other aspects (psychological, social and sexual) are not addressed.

Some studies in the literature show an overall improvement in the QOL of clinically treated women with endometriosis.^26^ ^34^ All of these studies were prospective and women were followed for at least 1 year. This makes us infer whether medical care could help improve the psychological aspects of QOL. The fact that our study evaluates women at just one moment of their lives may not reflect all aspects of QOL and, therefore, these women present a deterioration of their QOL mainly in relation to psychological aspects.

## Conclusion

Both questionnaires used in the current study addressed these issues and apparently produced similar results. It has been observed that medical treatment can help women improve in some aspects. Management is not effective at obtaining an excellent QOL, as endometriosis still exerts a significantly negative impact on a patient's QOL.
